# Non-genetic factors affecting the meat quality and flavor of Inner Mongolian lambs: A review

**DOI:** 10.3389/fvets.2022.1067880

**Published:** 2022-11-29

**Authors:** Yuning Liu, Runhang Li, Ying Ying, Yandong Zhang, Yiying Huang, Hongxin Wu, Kejian Lin

**Affiliations:** Laboratory of Grass Product Safety Risk Assessment of Ministry of Agriculture and Rural Affairs, Institute of Grassland Research, Chinese Academy of Agricultural Sciences, Hohhot, China

**Keywords:** meat quality, Inner Mongolian, lamb (meat), flavor, non-genetic factors

## Abstract

The Inner Mongolia Autonomous Region ranks first among the five major pastoral areas in terms of lamb breeding of China. The Inner Mongolia Autonomous Region has a vast territory, with many famous grasslands and thousands of forage plants and multiple local high-quality lamb breeds. After hundreds of years of artificial breeding and improvement, Mongolian sheep have developed many varieties. Different diets, feeding and treatment methods have effects on the production performance, lipid deposition and flavor composition of mutton sheep. Therefore, understanding the relationship among Inner Mongolian lamb, meat quality, and flavor will improve the production of high-quality mutton. The regulation of meat quality and flavor will have a profound impact on the deep processing and income-generating capabilities of mutton. Non-genetic factors affect the quality and flavor of mutton, which are more intuitive than genetic factors. In this review, we cover the contributions made by scientists to explore and improve the quality and flavor of Inner Mongolia lambs through non-genetic means, compare the differences between grazing and drylot-feeding in detail, and summarize some feed additives. We hope that based on our review, we can provide some inspiration to improve the meat quality of Mongolian sheep.

## Introduction

China is the world's largest producer of mutton. Among the five major pastoral areas in China, the Inner Mongolia Autonomous Region ranks first in the mutton industry in the country, accounting for 33% of the country's total mutton production (1). There are many famous grasslands in Inner Mongolia, such as Hulunbuir grassland, Xilin Gol grassland, Horqin grassland, Ulanchabu grassland, Ordos grassland, and Urad grassland. Thousands of pasture grasses grow on these grasslands, of which hundreds are of high feed value and palatability; especially fescue, wheatgrass, elm and wild oats, which are very suitable for feeding livestock (2). Therefore, the lamb produced in Inner Mongolia is pure and green, with superior quality, and is unanimously favored by consumers in China (1, 3).

According to geographical distribution and genetic relationship classification, there are Tibetan sheep, Mongolian sheep and Kazakh sheep in China. Mongolian sheep is a short fat-tailed sheep, which is the most abundant and widely distributed sheep breed in China, and is suitable for extensive management of nomadic grazing throughout the year (4). Mongolian sheep are distributed in northern China. They are obviously different from Tibetan sheep and Kazakh sheep in the Qinghai-Tibet Plateau and Xinjiang region in appearance (such as horn, head, and body shape). Studies have shown that Mongolian sheep in China evolved from wild argali in the mountains of Central Asia and spread mainly in northern rural areas. Mongolian sheep has strong adaptability to the ecological environment and is regarded as a valuable breed resource. Generally, it is bred purebred or crossed with foreign breeds to improve breed quality; it can also be used as a hybrid parent for commercial production (5). Since the 1960s, the production development trend of the international sheep breeding industry has shifted from “wool major, meat minor” to “meat major, wool minor.” In this context, to improve local Mongolian sheep, Inner Mongolia actively introduced Xinjiang fine wool sheep, Soviet Merino sheep, German Merino sheep, Du Po sheep, Caucasian Merino, Stavrop sheep, Shalisk sheep, tsigai sheep, Coryday sheep, and so on. The great development of the mutton industry in Inner Mongolia began in the early 1990s. With the weak domestic wool market, the demand for mutton gradually increased, and the demand for green and high-quality mutton increased significantly, which to a large extent promoted the development of the mutton industry in Inner Mongolia. The mutton industry has made great contributions to improving the dietary structure of residents, improving the physical fitness of the people, increasing the income of farmers and herdsmen, and promoting the adjustment of the production structure of agriculture, especially animal husbandry, and has gradually grown into a “sunrise industry” in Inner Mongolia's animal husbandry. Owing to the vast territory of the Inner Mongolia Autonomous Region, and based on the long-term natural and artificial selection of Mongolian sheep (6), there are many high-quality sheep in the area such as Ujumqin sheep, Sunite sheep, Hulunbuir sheep, Zhaowuda sheep, Bamai sheep etc. For more than 2000 years, with the free trade and the southward migration of the various tribes of the grassland, a large number of people have entered the area south of the Great Wall. Mongolian sheep have been artificially bred in the east and south of China, producing many excellent sheep breeds with distinctive characteristics, such as Hu sheep, Tong sheep, and small-tailed Han sheep (7).

Mutton is an indispensable source of high-quality protein in the human diet (8). The content of lysine, arginine, histidine, thiamine, riboflavin, and so on is higher than in other meats. Muscle fibers are delicate, soft, and moderately fat. Mutton has low cholesterol and high digestible protein content. The characteristic flavor of mutton is the result of the comprehensive effect of various flavor substances. In the mutton production process, in addition to genetic factors, variables such as livestock age, gender, castration, tail docking, feed additives, nutritional level of the diet, grazing and drylot-feeding also affect the composition and content of flavor substances in mutton to a certain extent (9). Therefore, understanding the relationship between Inner Mongolian lamb and its meat's quality and flavor will improve the production of high-quality mutton. The regulation of meat quality and flavor will have a profound impact on the deep processing and income-generating capabilities of mutton. Herein we review the contributions made by scientists to explore and improve the quality and flavor of Inner Mongolia lambs through non-genetic means, compare the differences between grazing and drylot-feeding in detail, and summarize some feed additives.

## Livestock age

The tenderness of mutton is greatly affected by age, but the change is small from birth to 1 year old. After sexual maturity of sheep, the increase of intramuscular fat slowed down with the increase of age, muscle fibers became significantly hard, and the tenderness was worse than that of lambs (10–16). Su and Zhang et al. compared the meat quality of 4, 6, 8, and 12-month-old Bamai sheep, small-tailed Han sheep, and Sunite sheep. The results showed that Bamai sheep reached the best slaughter time at the age of 6 months and had the best eating quality. Also, as the age in months increases, the growth rate of its high-quality meat pieces is also greater than that of other meat pieces. Therefore, Bamai sheep has superiority and good meat production performance in terms of slaughter performance and carcass weight (17–21). Su et al. pointed out that the types of Sunite sheep's muscle fiber (*longissimus thoracis, biceps femoris*, and *triceps brachii*) are mainly glycolytic type II B fibers. With the increase of months, the muscle fiber has a trend of I → IIA → IIB. The diameter and cross-sectional area of muscle fiber gradually increase with the number of months, but the increase is slower after the age of 6 months (22). Wang et al. pointed out that under natural grazing conditions, with the increase in months, the muscle fiber diameter and perimysium of Ujumqin sheep showed a steady increasing trend from 1 to 9 months of age, and the increasing trend decreased by 12 months of age; however, the endomysium in the connective tissue of the *semitendinosus* and *biceps femoris* tends to thicken (23). Zhang et al. detected 31 fatty acids in the *semitendinosus* muscle of Ujumqin sheep. As the age in months increased, UFA showed an upward trend, while the relative expression of SFA, MUFA, PUFA and PPARγ, and FAS genes was negatively correlated (24). As the age increases, the flavor of mutton becomes richer, gamy odor will also increase, and the tenderness will decrease. Outside the Inner Mongolia Autonomous Region, lambs <1 year old sell best (especially lambs around 6 months old). However, consumers in the region are more tolerant of lamb of different ages, while the standard of delicious mutton is more diversified, and lamb of different ages appears on the menu. Many local herdsmen prefer 2-year-old or even 3-year-old mutton, and there is little research on the meat quality of older sheep.

## Gender and castration

Improving meat quality through gender and castration mainly depends on changing the secretion of sex hormones in the body to control fat deposition and volatile substances. The difference in gender mainly affects the texture and flavor of mutton. The texture of ram meat is relatively rough, hard, and with a special smell. In addition, rams have lower intramuscular fat content than ewes, but their feed conversion rate is about 13% higher than that of ewes (25, 26). To study the effect of gender on the quality of Bamai sheep, Zhang et al. selected 4, 6, 8, and 12-month-old Bamai sheep, small tail Han sheep, and Sunite sheep to compare their slaughter performance, carcass weight, and meat quality of *longissimus thoracis, biceps femoris*, and *triceps brachii*. The shear force value of the *biceps femoris* of ewes aged 4–6 months is significantly lower than that of rams of the same breed (27–30). Liu et al. pointed out that the carcass weight of Bamei rams is better than that of Bamei ewes and has better meat production performance, whereas the eating qualities of ewes, such as tenderness and cooked meat rate, are better than those of rams. The measurement results of the electronic tongue show that the types of flavor substances in ewes are more abundant than those in rams. Rams have higher content of 2,3-octanedione and capric acid; the content of 3-hydroxy-2-butanone and benzaldehyde in ewes is higher. Among the fatty acids, rams have higher content of c-OA and eicosadienoic acid; the content of PA and OA in ewes is higher. Rams have a higher ratio of PUFA to SFA. In general, the carcass weight of rams and the nutritional value of fatty acids are better than those of ewes, but the eating quality of ewes is better than that of rams (31). Gao and Ji et al. pointed out that the bone weight and bone-to-flesh ratio of Sunite rams are significantly higher than in ewes, and the net meat weight, net meat percentage, and crude fat content of ewes are significantly higher than in rams. The drip loss rate of ewes is significantly lower than that of rams. The intramuscular water content of rams is significantly higher than that of ewes. This conclusion was confirmed in Liu et al.'s (25) study on 8-month-old Sunite sheep of different genders. The ratio of essential amino acids in muscles of ewe, the ratio of SFA in subcutaneous fat, and the ratio of monounsaturated fatty acid (MSFA) in tail fat are significantly higher than in rams. Gender has no significant effect on the amino acid content of precursors that form meat flavor. The ratio of PUFA in intramuscular fat of rams is significantly higher than that of ewes. In addition, H-FABP is positively correlated with intramuscular fat content, which leads to significantly higher intramuscular fat of ewes than that of rams (32, 33). Whether it is a sheep farm or herdsmen in the region, they have reached a consensus on using rams to produce mutton. Most of the time, ewes are most used for fertility and lactation. Because ewes are richer in amino acids and fatty acids, local herdsmen also choose ewes to stew and drink soup.

Castration is a traditional practice used in most countries to improve meat quality and reduce aggressive behavior from livestock. Previous studies have shown that castration changes the lipid accumulation of ruminants and changes the characteristics of volatile compounds (34). Castration does have an effect on raw meat fatty acids. Castrated rams are called wethers. Studies have reported that, compared with wethers, rams grow faster, have higher feed utilization rates, and have higher carcass lean meat ratios, which are caused by the stimulation of hormones, especially testosterone. Experiments have proved that under the same feeding and management conditions, the average daily gain of rams is 230 g, while that of wethers is 200 g. The feed conversion rate of rams is 12%−15% higher than that of wethers, but the average slaughter rate of rams (49.6%) is lower than that of wethers (51.3%), and the tenderness of ram meat is not as good as that of wethers. As for the other edible characteristics of the two, there is no significant difference (35). Li et al. pointed out that castration significantly changed the content of water-soluble flavor precursors (such as flavor amino acids, 5-phosphate ribose, and hypoxanthine) and increased the content of important fat-soluble flavor precursors (such as phospholipids and glycerides); the content of volatile substances (such as 1-octen-3-ol and hexanal) increased significantly, and the fatty aroma of mutton and the aroma of grass became more obvious (36). The sheep farms and herdsmen in the region have not reached a consensus on castration. Based on the consensus of using rams to produce lamb, the time to castration is around 2–3 weeks or 13–16 weeks of age. Castrated rams did gain a good reputation in the Inner Mongolia Autonomous Region. The growth rate, meat quality, and feed conversion ratio of castrated ewes have been improved, but there are very few studies on castrated ewes.

## Tail docking

In recent years, with the improvement of breeding technology, the animal production cycle is increasingly shortened, the level of animal production is increasingly improved, and the fat deposit in the animal body is gradually increasing. Excessive fat deposition not only affects the carcass weight of animals but also seriously affects the processing of animal products. More concerningly, eating too much fat will increase the incidence of some diseases and cause energy waste. To further tap the production potential of livestock resources, changing the form of animal fat distribution, reducing carcass fat deposition, improving meat quality, and improving overall economic benefits have become the focus of attention of researchers and livestock workers (4). Under the intensive production model, early tail docking plays an increasingly important role in fattening animals, and the development of early tail docking of animals has a crucial impact on animal growth and development, meat production performance, and meat quality (37, 38). Ran et al.'s research showed that the lean meat rate and eye muscle area of the tail-docking sheep have been greatly improved, and the fat deposits are transferred from the tail to the intermuscular and subcutaneous, thereby improving the meat and carcass weight. This shows that tail docking is an effective way to improve the economic benefits of sheep raising. Liu et al. (39) showed that tail docking has no significant effect on the cooked meat rate, color, pH, and marbling of Lanzhou big-tail sheep, but it has a tendency to reduce water loss and shear force value and increase pH. Tail docking has no significant effect on the meat quality of Mongolian sheep, as proven by Marai et al. Zhou et al. showed that tail docking had no effect on the live mass and carcass mass of Lanzhou big-tail sheep and Mongolian sheep. On the contrary, the slaughter performance and meat quality of Lanzhou big-tail sheep have improved, even better than Mongolian sheep (40). The aforementioned studies have shown that the fat in the tail of the animal has a tendency to transfer to the muscles and subcutaneously after the early tail docking, thereby improving the carcass characteristics, meat quality, and palatability. Tail docking can indeed improve meat quality and improve feed conversion, but not all sheep farms and herders do tail docking of mutton sheep. Sheep tail is an important part for extracting suet, and suet is also an important cooking material (41). At the same time, many people in the region (especially herders) consider sheep tails to be a delicacy.

## Grazing and drylot-feeding

Different feeding methods directly affect the growth rate, weight gain, and meat quality. The common feeding methods in actual production include grazing, semi-drylot-feeding (grazing and supplementary feeding), and whole-drylot-feeding (42). As the most traditional lamb production method in many countries, natural grazing is the one with the lowest investment, the highest animal welfare, and the best ecological benefits. Consumers generally believe that mutton produced under natural grazing conditions is “healthier, more nutritious, and more natural,” which means that grazing is the most demanding way of producing lamb (2). In winter and spring, owing to the reduced palatability and nutritional value of pasture, grazing sheep have insufficient nutrient intake, slow growth, and low production efficiency. Appropriate supplementary feeding can meet the nutritional requirements for optimal growth, carcass, and meat quality production (43–45).

Jin et al. conducted a long-term study on the meat quality of Sunite sheep raised in house and grazing, exploring the effects of feeding methods on the growth performance, meat quality, flavor, and fatty acid composition of Sunite sheep (Tables 1, 2) (46–49). Sunite sheep (10 sheeps per group) raised in house or grazing, and slaughtered at 12 months of age for the collection of muscle samples; the growth status, pre-slaughter weight, carcass weight, protein, ash, thiamine, reducing sugar content, pH, and tenderness of the *longissimus thoracis* muscle of the grazing group were significantly higher than those of the drylot-feeding group; the fat content, slaughter, net meat rate, and redness value of the drylot-feeding group were higher than those of the grazing group (50). In addition, after 45 min of slaughter, the pH value of mutton in the drylot-feeding group was significantly higher than that of the grazing group, and after 24 h, there was no significant difference in the two groups. The grazing group had higher levels of skeletal muscle satellite cell specific markers (Pax7), myogenic determinant (MyoD), and PPARγ, which led to the development of skeletal muscle satellite cells in the direction of myogenesis instead of adipogenesis. In addition, endurance exercise changes the components of the myosin heavy chain from oxidized isomers to glycolytic type, resulting in a drop in pH and a change in color (46). Meanwhile, the concentration, vitality and mRNA expression of adenylate-activated protein kinase (AMPK), the concentration of carnitine palmitoyl transferase 1 (CPT1), L^*^, b^*^ and shear force values in the *biceps femoris* of the grazing group were significantly higher than those of the drylot-feeding group; the activity and mRNA expression of ACC and intramuscular fat content of *biceps femoris* in the grazing group were significantly lower than those in the drylot-feeding group (51, 52). Therefore, to a certain extent, the AMPK-ACC-CPT1 pathway can be activated by grazing, thereby reducing intramuscular fat deposition, increasing the L^*^ and b^*^ values of mutton, reducing tenderness, and affecting meat quality (53). The expression of miRNAs in the grazing group was higher than that in the drylot-feeding group. The expression of miR-1 in the grazing group was significantly positively correlated with body height, carcass weight, net meat weight, and net meat rate. The expression of miR-133 was significantly positively correlated with net meat weight, while the expression of miR-128 in the drylot-feeding group was significantly negatively correlated with net meat rate, indicating that different feeding conditions have an impact on the expression of miRNAs and further affect slaughter performance (54). miR-30a has a significant negative correlation with the redness value. Different feeding methods have an impact on the expression of miR-128, miR-486, miR-30a, and miR-223 and further affect the quality of meat (55). The diameter and cross-sectional area of type I and type IIB *longissimus thoracis* muscle fibers in the grazing group were significantly smaller than those in the drylot-feeding group. At the same time, the ratio and area ratio of type I and type IIA muscle fibers of *longissimus thoracis* in the grazing group, and the mRNA expression of MyHC I and MyHC II a genes, were significantly higher than those in the drylot-feeding group. The ratio of type IIB muscle fibers and the mRNA expression of MyHC type IIb genes is the opposite (Table 3) (56). The UFA content of the grazing group was significantly higher than that of the drylot-feeding group, especially CLA, ALA, EPA, and DHA. In contrast, the PA concentration in the subcutaneous fat tissue of the drylot-feeding group was significantly increased. The expression levels of superoxide dismutase (SOD), catalase (CAT), glutathione peroxidase (GPx), fatty acid desaturase 1 (FADS1), fatty acid desaturase 2 (FADS2), PPARγ, and lipoprotein lipase (LPL) gene of tail fat and kidney fat in the grazing group were significantly higher than those in the drylot-feeding group (Table 4) (57, 58). The FADS1, FADS2, and Elove5 gene expression increased significantly in the grazing group, indicating that the high expression of FASD gene is beneficial to the deposition of ALA, DHA, and EPA in the grazing group (48). The expression levels of lipoxygenase (LOX), stearoyl-CoA desaturase (SCD), and acetyl-CoA carboxylase α genes of the *longissimus thoracis* muscle in the drylot-feeding group were significantly higher than those in the grazing group. The expression of SCD gene in the drylot-feeding group increased significantly, indicating that drylot-feeding is beneficial to the synthesis of OA. At the same time, rumen bacteria play a key role in feed degradation and productivity. Feeding methods have impacts on the rumen microbial population and fatty acid composition of mutton. The abundance of Butyrivibrio_2, Saccharofermentans, and Succiniclasticum in the grazing group was higher than that in the drylot-feeding group, while the abundance of RC9_gut_group was lower (47). The n-3PUFA content of the grazing group was higher than that of the drylot-feeding group. In addition, the content of ALA and CLA was positively correlated with the abundance of Butyrivibrio_2. In addition, after 3 months of storage, the thiobarbituric acid value of the grazing group was significantly lower than that of the drylot-feeding group, indicating that the mutton of the grazing group had higher antioxidant capacity (59). Of course, the oxidative stability of mutton in the grazing group was significantly higher than that in the drylot-feeding group; this may be related to the greater exercise of the grazing group, which increased the activity of antioxidant enzymes in its muscles (46). Therefore, grazing conditions are more conducive to the deposition of energy storage fat, and the degree of lipid oxidation in the drylot-feeding group is more serious than that of the grazing group (60). The main volatile compounds in lamb are hexanal, nonanal, 1-octen-3-ol, and 2,3-octanedione. The content of 1-octen-3-ol and 2,3-octanedione in the grazing group was significantly higher than that of the house-fed group, while the content of nonanal was significantly lower than that of the house-fed group (57, 61). Owing to the higher expression levels of PPARγ and ACC genes in the grazing group, more fatty acids are synthesized and deposited in the mutton, and the high expression of LOX genes is activated, which promotes the oxidation of fatty acids to produce a large amount of aldehydes and alcohol volatile substances, giving excellent flavor quality of grazing lamb (61). The umami and salty tastes of meat in the grazing group were higher than those in the drylot-feeding group, and the bitterness and astringency of meat were lower than those in the drylot-feeding group. There was a significant positive correlation between the expression of adenylate succinate lyase and hypoxanthine nucleotide cyclohydrolase genes and the IMP content of grazing group. When the expression of umami substance-related genes is high, the content of umami substance in meat also increases (62). Although the drylot-feeding group has advantages in slaughter and processing, the grazing group is conducive to the accumulation of protein, minerals, reducing sugars, and other nutrients in the Sunite sheep, so that the nutrient content of the mutton is richer and the quality is better. In production, different feeding modes can be selected according to different uses. A series of research results of Jin's team showed that feeding methods can become an important tool to change the quality of animal meat by changing the gene expression of enzymes involved in fat metabolism.

**Table 1:** Effects of feeding regimes on slaughter performance, carcass characteristics, meat quality, fatty acids, expression of skeletal muscle-related genes and miRNAs of Sunite sheep^*^.

**Item**	**Grazing**	**House-feeding**	**References**
Body length/cm	59.70 ± 2.79	64.90 ± 3.17	(59)
Body height/cm	59.40 ± 2.17	65.30 ± 2.58	(59)
Chest circumference/cm	79.15 ± 1.76	87.20 ± 3.05	(59)
Body weight/kg	38.78 ± 3.43	48.13 ± 3.52	(59)
Thigh bone weight/kg	1.19 ± 0.11	1.19 ± 0.09	(59)
Rib weight/kg	0.84 ± 0.13	0.89 ± 0.08	(59)
Spine weight/kg	1.76 ± 0.20	1.92 ± 0.15	(59)
Total bone weight/kg	3.78 ± 0.34	34.01 ± 0.28	(59)
Net meat weight/kg	13.04 ± 1.30	19.88 ± 2.63	(59)
Ratio of bone to flesh	0.29 ± 0.02	0.21 ± 0.03	(59)
Slaughter rate	0.46 ± 0.02	0.51 ± 0.04	(59)
Carcass length/cm	62.50 ± 1.58	61.30 ± 1.63	(59)
Carcass depth/cm	21.50 ± 1.17	23.50 ± 1.76	(59)
Carcass weight/kg	17.76 ± 1.45	24.82 ± 3.88	(59)
Eye muscle area/cm^2^	16.76 ± 3.09	20.27 ± 2.23	(59)
Protein/%	20.04 ± 1.25	20.39 ± 0.9	(59)
Moisture/%	74.34 ± 0.14	74.74 ± 0.19	(59)
Ash/%	1.06 ± 0.16	0.99 ± 0.09	(59)
Fat/%	3.30 ± 0.93	5.25 ± 0.45	(59)
Cholesterol (g/100 g)	0.46 ± 0.04	0.49 ± 0.05	(59)
L^*^ (Subcutaneous fat)	73.19 ± 3.17	73.31 ± 3.48	(59)
a^*^ (Subcutaneous fat)	7.49 ± 1.25	4.62 ± 1.09	(59)
b^*^ (Subcutaneous fat)	11.37 ± 0.82	7.26 ± 1.51	(59)
Shear force/N (Subcutaneous fat)	47.94 ± 6.52	54.59 ± 4.62	(59)
Capric acid (C_10:0_)/%	0.08 ± 0.02	0.11 ± 0.02	(59)
Lauric acid (C_12:0_)/%	0.15 ± 0.05	0.15 ± 0.03	(59)
Myristic acid (C_4:0_)/%	1.80 ± 0.34	1.86 ± 0.24	(59)
Myristoleic acid (C_4:1_)/%	0.30 ± 0.10	0.21+0.06	(59)
Palmitic acid (C_16:0_)/%	19.83 ± 1.28	21.16 ± 0.91	(59)
Palmitoleic acid (C_16:1_)/%	2.01 ± 0.20	1.99 ± 0.21	(59)
Stearic acid (C_18:0_)/%	16.64 ± 0.92	15.41 ± 1.57	(59)
Oleic acid (C_18:1_)/%	38.28 ± 2.82	42.17 ± 1.39	(59)
Linoleic acid (C_18:2_)/%	7.46 ± 1.03	8.33 ± 1.29	(59)
Arachidic acid (C_20:0_)/%	0.12 ± 0.04	0.12 ± 0.02	(59)
γ-Linolenic acid (C_18:3n6_)/%	0.11 ± 0.02	0.07 ± 0.01	(59)
α-Arachidic acid (C_18:3n3_)/%	2.01 ± 0.35	0.34 ± 0.08	(59)
CLA_cis − 9, trans − 11_/%	0.83 ± 0.05	0.41 ± 0.81	(59)
cis-11,14-Eicosadienoic acid (C_20:2_)/%	0.10 ± 0.04	0.07 ± 0.03	(59)
Behenic acid (C_22:0_)/%	0.54 ± 0.35	0.67 ± 0.20	(59)
Eicosatrienoic acid (C_20:3n6_)/%	0.28 ± 0.06	0.20 ± 0.04	(59)
Arachidonic acid (C_20:4n6_)/%	2.65 ± 0.72	3.03 ± 0.80	(59)
Tetracosanoic acid (C_24:0_)/%	0.07 ± 0.01	0.09 ± 0.04	(59)
EPA (C_20:5_)/%	0.77 ± 0.16	0.09 ± 0.05	(59)
DHA (C_22:6_)/%	0.22 ± 0.08	0.05 ± 0.01	(59)
SFA/%	41.04 ± 2.06	41.05 ± 1.27	(59)
MUFA/%	42.46 ± 2.82	45.63 ± 1.51	(59)
PUFA/%	16.13 ± 1.54	11.69 ± 1.59	(59)
n-6 PUFA/%	12.19 ± 2.03	10.72 ± 0.54	(59)
n-3 PUFA/%	3.01 ± 0.57	0.48 ± 0.12	(59)
Tenderness/N	74.097 ± 4.557	72.185 ± 3.190	(55)
L^*^	25.558 ± 0.701	27.615 ± 1.221	(55)
a^*^	17.166 ± 1.580	22.770 ± 1.667	(55)
b^*^	13.950 ± 0.894	8.413 ± 0.838	(55)
pH_0_	6.303 ± 0.137	6.476 ± 0.075	(55)
pH_24_	5.196 ± 0.059	5.438 ± 0.066	(55)
Cooked meat rate	61.000 ± 0.010	61.300 ± 0.009	(55)
Cooking loss	39.000 ± 0.011	38.700 ± 0.009	(55)
Hexokinase activity (U/g)	47.36 ± 9.06	53.20 ± 9.69	(51)
Lactic acid (mmol/g)	1.23 ± 0.14	1.45 ± 0.08	(51)
Muscle glycogen (mg/g)	5.87 ± 0.84	5.72 ± 0.70	(51)
Shear force (kg/cm^2^)	41.84 ± 5.50	32.86 ± 4.16	(51)
*miR-30a*	√		(55)
*miR-223*	√		(55)
*miR-128*	√		(55)
*miR-486*	√		(55)
*miR-1*	√		(54)
*miR-206*	√		(54)
*miR-128*	√		(54)
*miR-486*	√		(54)
*miR-133*	√		(54)
*miR-23a*	√		(54)
*MyHC I*	√		(56)
*MyHC II a*	√		(56)
*MyHC II b*		√	(56)
*MyHC II x*		√	(56)
*PRKAA1*		√	(51)
*PRKAA2*	√		(51)
*PRKAG3*		√	(51)

* Average values for different muscles if not specified.

√The value of the item is higher under this feeding regime than the another feeding regime (the specific number is not given in the original text).

**Table 2:** Effects of feeding regimes on meat quality, nutrients, trace elements and umami substances of *Triceps brachii* (TB), *Longissimus thoracis* (LT), *Biceps femoris* (BF) of Sunite sheep.

**Item**	**Grazing**			**House-feeding**			**References**
	**TB**	**LT**	**BF**	**TB**	**LT**	**BF**	
Ash (g/100 g)	1.090 ± 0.091	0.864 ± 0.091	1.092 ± 0.151	1.012 ± 0.142	0.976 ± 0.067	0.911 ± 0.124	(50)
Fat (g/100 g)	3.301 ± 0.931	4.063 ± 0.482	2.778 ± 0.461	5.254 ± 0.456	6.826 ± 0.620	6.674 ± 0.521	(50)
Protein (g/100 g)	20.39 ± 0.900	–	21.14 ± 1.945	20.040 ± 1.254	–	20.080 ± 0.445	(50)
Thiamine		√		√		√	(50)
pH_0_	6.240 ± 0.096	6.405 ± 0.206	6.350 ± 0.087	6.509 ± 0.162	6.790 ± 0.021	6.559 ± 0.099	(50)
pH_24_	5.907 ± 0.060	5.615 ± 0.037	5.738 ± 0.035	5.844 ± 0.119	5.604 ± 0.046	5.858 ± 0.157	(50)
Reducing sugar (mg/g)	2.342 ± 0.337	3.836 ± 0.139	2.333 ± 0.382	1.950 ± 0.387	2.780 ± 0.208	1.908 ± 0.251	(50)
Ca (mg/100 g)	4.248 ± 0.287	4.228 ± 0.194	4.170 ± 0.332	4.392 ± 0.275	4.337 ± 0.186	4.261 ± 0.281	(50)
K (mg/100 g)	230.610 ± 7.210	238.460 ± 10.920	239.130 ± 7.150	205.480 ± 9.740	236.050 ± 12.030	236.520 ± 5.290	(50)
Na (mg/100 g)	383.005 ± 11.641	371.029 ± 19.861	393.370 ± 11.127	391.344 ± 15.545	359.086 ± 15.738	376.082 ± 13.466	(50)
Mn (mg/100 g)	0.122 ± 0.003	0.123 ± 0.005	0.121 ± 0.003	0.122 ± 0.004	0.124 ± 0.005	0.121 ± 0.004	(50)
Zn (mg/100 g)	8.530 ± 0.590	5.417 ± 0.374	8.455 ± 0.888	9.052 ± 0.542	5.469 ± 0.459	9.413 ± 0.844	(50)
Cu (mg/100 g)	0.333 ± 0.014	0.337 ± 0.025	0.331 ± 0.017	0.346 ± 0.020	0.346 ± 0.028	0.331 ± 0.013	(50)
Fe (mg/100 g)	4.320 ± 0.006	4.415 ± 0.423	4.560 ± 0.302	5.457 ± 0.818	5.272 ± 0.129	5.603 ± 0.195	(50)
IMP	1.72 ± 0.16	1.70 ± 0.17	1.50 ± 0.14	1.46+0.14	1.46 ± 0.16	1.48 ± 0.15	(62)
INO	0.52 ± 0.04	0.68 ± 0.09	0.52 ± 0.07	0.39 ± 0.04	0.48 ± 0.11	0.47 ± 0.03	(62)
HYP	0.36 ± 0.05	0.43 ± 0.07	0.39 ± 0.04	0.35 ± 0.03	0.46 ± 0.20	0.31 ± 0.04	(62)
AMP	0.115 ± 0.010	0.119+0.021	0.107 ± 0.013	0.072 ± 0.008	0.168 ± 0.023	0.084 ± 0.025	(62)
ADP	0.081 ± 0.009	0.100 ± 0.017	0.075 ± 0.014	0.076 ± 0.004	0.114 ± 0.016	0.078 ± 0.012	(62)

√ The value of the item is higher under this feeding regime than the another feeding regime (the specific number is not given in the original text).

IMP, inosine monophosphate; INO, inosine; HYP, hypoxanthine; AMP, adenosinemonophosphate; ADP, adenosinediphosphate.

**Table 3:** Effects of feeding regimes on muscle fiber types of *Longissimus thoracis* of Sunit sheep (56).

**Item**	**Grazing**			**House-feeding**		
	**I**	**IIA**	**IIB**	**I**	**IIA**	**IIB**
Diameter				√	√	√
Cross-sectional area				√	√	√
Quantity	√	√				√
Area ratio	√	√				√

√ The value of the item is higher under this feeding regime than the another feeding regime (the specific number is not given in the original text).

**Table 4:** Effects of feeding regimes on the expression of fat metabolism genes in Intramuscular fat (IF), Tail fat (TF), Kidney fat (KF) of Sunite sheep (58).

**Item**	**Grazing**			**House-feeding**		
	**IF**	**TF**	**KF**	**IF**	**TF**	**KF**
SCD				√	√	√
FADS1	√	√	√			
FADS2	√	√	√			
Elove5	√	√	√			
ACC		√		√		√
LPL	√	√	√			
FABP4	√	√	√			
CPT1	√		√		√	
PPARy	√	√				√

√ The value of the item is higher under this feeding regime than the another feeding regime (the specific number is not given in the original text).

Qian et al. pointed out that drylot-feeding can provide higher dietary nutrient levels for lamb, and the house-fed sheep has less exercise, low energy consumption, fast weight gain, and better meat production performance. The grazing sheep will walk a longer distance to eat, increase the amount of exercise, and consume a lot of energy, resulting in less fat in their muscles and more lean meat (63). Hamdi et al. pointed out that the lean meat of grazing sheep was significantly higher than that of drylot-feeding sheep, and their meat quality was tender and juicier (64). Ponnampalam et al. pointed out that the n-3 PUFA content and PUFA:SFA ratio in grazing lamb meat were higher than those of the drylot-feeding lambs, and the n-6:n-3 ratio was lower than that of the drylot-feeding lambs. During grazing, the forages eaten by lamb can promote the growth of fiber-decomposing microorganisms responsible for the hydrogenation process in the rumen, thereby increasing the content of muscle CLA and its precursors (65). Guo et al. studied the content of mineral elements in the blood of grazing Sunite sheep in Fuyuan District (mineral element deficiency area) in Sunite Left Banner, Inner Mongolia. The results showed that the content of Cu, Zn, Ca, P, and Fe in the blood of grazing sheep was significantly lower than that of shed sheep (66). Hou et al. pointed out that the collagen content and thermal solubility of Ujumqin sheep slaughtered in summer are higher than in winter; the content of pyridinoline is higher in winter than in summer; and the content of collagen and pyridinoline in the *semitendinosus* and *longissimus thoracis* muscle of the grazing group and the heat denaturation temperature were higher than those of the drylot-feeding group (67). Yang et al. pointed out that the feeding method has changed the characteristics of the *biceps femoris* muscle of Tan sheep and has an impact on the muscle ultrastructure and protein degradation during the maturation process after slaughter. Drylot-feeding increases the fiber density of the *biceps femoris* muscle, reduces the diameter and cross-sectional area of the muscle fiber, and reduces the shear force value of the muscle; in addition, owing to the increase in the proportion of type II muscle fibers, drylot-feeding increases the growth rate of muscle myofibril fragmentation index, sarcoplasmic protein solubility, and protein degradation rate after slaughter, accelerates the maturation process after slaughter, and improves the tenderness of the biceps muscle (68). Xie et al. (69) pointed out that compared with grazing, supplementary feeding of Hulunbuir lamb can significantly increase its fattening performance and carcass weight. Chen et al. (70) pointed out that the cooking loss rate of mutton was slightly lower and the tenderness was slightly higher than that of grazing group. Wang et al. pointed out that different grazing time has a certain impact on animal performance and fatty acid composition. When the grazing time was 8 h/d and 2 h/d, lamb body weight, carcass weight, and intramuscular fat content were higher. Sheep with longer grazing time and less concentrated feed accumulate more CLA and n-3PUFA, and the n-3/n-6 ratio is higher. It is recommended to use 8 h/d and supplementary feeding to maintain the meat quality (60). Hou et al. pointed out that grazing made the expression of myosin heavy chain 1 (MyHC I) gene significantly higher than that of drylot-feeding, while the expression of MyHC IIa and MyHC IIb genes was significantly lower than that of drylot-feeding. In addition, compared with the drylot-feeding group, the AMPK activity and the expression of AMPK α2 and PGC-1α genes in the grazing group were significantly increased. Muscle fiber composition is one of the key meat quality differences caused by different feeding schemes. AMPKα2 and PGC-1α are considered to be two key factors regulating Mongolian sheep muscle fiber types (71). Wang et al. pointed out that feeding methods have significantly changed the metabolic homeostasis of Mongolian sheep. There is a substantial correlation between several gut microbiota and the composition of fecal and plasma metabolites, especially metabolites involved in the metabolism of butyric acid, ALA, and L-tyrosine. Owing to the high energy level of the diet and less exercise, the drylot-feeding group had more intramuscular fat deposits, finer muscle fibers, and lower shear force value than the grazing group, but better tenderness (8). Whether inside or outside the Inner Mongolia Autonomous Region, grazing sheep is always more popular than house feeding. For reasons of policy and economic interest, semi-house feeding and appropriate supplementary feeding are increasingly accepted. There are also fake grazing sheep on the market, and the traceability of grazing sheep has become an urgent problem to be solved (72). At the same time, the Inner Mongolia Autonomous Region has many grasslands with different climatic conditions, all of which have formed unique grazing varieties. Alxa League belongs to desert-type grassland, and local grazing sheep need to walk a long distance to obtain enough forage. While some grasslands in eastern Inner Mongolia are rich in water and grass (such as Hulunbuir grassland), local grazing sheep only need proper exercise to obtain a large amount of fresh forage. Different grazing environment and amount of exercise have obvious effects on the cross-sectional area, tenderness, flavor and taint of grazing sheep (73). The distinction between different grassland sheep is also an important issue.

## Nutritional level of the diet

Nutrition level and diet composition increase the difference in carcass composition. The addition of appropriate protein in the feed can increase the amount of fat deposits in the body and improve the quality of meat (74–77). Experiments have shown that the taste of certain mutton is related to aromatic wild pastures (such as white clover, *Alfalfa*, rape, and oats). After sheep have eaten odorous forages, and are then fed without odorous forages for 7–14 days, the odor can be eliminated (35). Li et al. pointed out that there are also differences in the flavor of lamb when grazing on different types of pastures. For example, lamb grazing on leguminous pastures (such as *Alfalfa* and white clover) produce significantly higher volatile indole content in the rumen than those grazing on other types of pastures, so their flavor is poor (61). Larick et al. (78) pointed out that there is a strong positive correlation between δ-14 lactones and δ-16 lactones and the amount of cereal diets fed, and that these two lactones can be used as indicators for cereal feeding. Sebastian et al. found that sheep fed with cereal diets had higher OA content; lower CLA content; and higher 4-heptenal, 2,4-heptadienal, and 2,6-nonadienal (decomposed from LA) content in grazing sheep fat, and feeding with concentrated feed increased the content of hexanal, heptanal, 2,4-decadienal, and so on (from OA) in sheep fat. They also found that 4-heptenal can be used as a characteristic product of grazing (79). Chen et al. pointed out that whole-plant corn silage can significantly improve the growth performance, slaughter performance, and immune function of lamb (80). The research results of Zhao et al. (81) showed that whole-plant corn silage reduced the average daily dry matter intake, feed-to-weight ratio, *longissimus thoracis* fat content, and drip loss rate of mutton. Tian et al. (82) pointed out that different feeding levels have no significant effect on the weight gain ratio, slaughter rate, and meat quality of sheep, but free intake can significantly increase sheep's daily gain, intramuscular fat content, and muscle amino acid content. Guo and Zhao pointed out that replacing corn and soybean with the same amount of concentrate supplement can increase the protein utilization efficiency of Sunite ewes during the prelactation period and improve their negative balance of energy and calcium and phosphorus nutritional status. Further increase in the proportion of concentrate fed to promote weight recovery in ewes, increase the serum Ca, Cu, Zn, and Se content, and reduce the concentration of urea nitrogen and β-hydroxybutyric acid. Forage feeding also increased the expression of LPL, PPARγ, SCD, FADS1, and Elove5 in the subcutaneous adipose tissue, but the feeding regimen did not change the expression of FASN and CPT1 (83, 84). Du and Zhou et al. pointed out that the supplementary feed of grass pellets has little effect on the growth performance of Ujumqin sheep, feeding of natural grass pellets can meet the nutritional needs of livestock, has great potential in promoting the production performance, slaughter performance, and meat quality of Ujumqin sheep, and can effectively optimize the flavor and quality of mutton (85, 86). Cui et al. pointed out that low levels of protein or energy inhibited the growth performance and rumen development of lambs. The low energy level significantly reduced the content of volatile fatty acids. The relative abundance of Cellulomonas was increased at the phylum level, and the relative abundances of Vibrio bovis and Vibrio procerata were increased at the genus level. The authors significantly correlated 14 genus with the nutritional level in the rumen (87). Nutrient level is a very broad concept, and increasing the proportion of protein in the diet is just one of them. Like humans, lambs also need to eat freely, with more plant variety, proper exercise and supplemental feed.

## Feed additives

Feed additives can add nutrients necessary for livestock to the feed and have achieved the effect of strengthening the nutritional value of basic feed, improving animal production performance, ensuring animal health, saving feed costs, and improving the quality of animal products. Feed additives are generally protein, specific amino acids, unsaturated fatty acids, antioxidants, and natural products (88–92).

### Allium mongolicum

*Allium mongolicum* is a plant of the genus *Allium* with many biological activities. As a characteristic plant in desert grassland and sand dunes, *A. mongolicum* has strong drought and cold resistance. Its main distribution areas include Xinjiang, Qinghai, Gansu, and western Inner Mongolia. *Allium mongolicum* has a unique flavor and rich nutrition, rich in nutrients such as protein, amino acids, fats, minerals, trace elements, polysaccharides, and flavonoids. At the same time, eating it can lower blood pressure, reduce appetite, and improve immunity. It has anti-oxidation, anti-aging, antibacterial, and antiviral effects and is known as “Ganoderma lucidum in vegetables.” *Allium mongolicum* is also a forage that grows widely in the grasslands of Inner Mongolia and is a favorite food for sheep. According to many years of experience of local herders, the growth rate of sheep after eating *A. mongolicum* is accelerated, the incidence of disease is reduced, and the quality and flavor of meat can be significantly improved (93–95). Ao et al. have conducted long-term research on the application of *A. mongolicum* to the mutton industry (96, 97). The addition of *A. mongolicum* polysaccharides and *A. mongolicum* total flavonoids in the diet proves that *A. mongolicum* and its extracts can effectively improve the growth performance, antioxidant status, and immune response of lamb, while improving meat quality. It also affects the composition and content of fatty acids and flavor substances in muscles; in particular, the main flavor substances of *A. mongolicum* have a strong correlation with MSFA, and long-term intake will not cause kidney and liver diseases (98, 99). The addition of scallion flavonoids (22~33 mg/kg) in the diet can significantly improve the production performance of lamb and the expression of β-defensin-1 (sBD-1) and β-defensin-2 (sBD-2) genes in the intestinal tissues (100). Further, *A. mongolicum* polysaccharide can affect the proliferation of lymphocytes while releasing nitric oxide and inducible nitric oxide synthase (iNOS), indicating that *A. mongolicum* polysaccharide has an immunomodulatory effect on sheep peripheral blood lymphocytes (101).

### Selenium

Selenium is an important element in the diet of humans and livestock (102, 103). The selenium in livestock diets is usually provided by plant feeds, and the content of selenium in plant feeds varies greatly among regions. The selenium content in diets is often insufficient for animals, and selenium supplements from other sources need to be added to meet the demand (104, 105). Sodium selenite, yeast selenium, selenomethionine, selenocysteine, and selenium polysaccharides are commonly selenium-containing feed additives in animal husbandry production (106). Jia et al. pointed out that the addition of yeast selenium (Se 0.25~2.00 mg/kg) in the diet has no adverse effects on Tan sheep's growth performance, blood routine parameters, selenoprotein gene expression, tissues, and organs. It is safe to feed Tan sheep when the dietary selenium content reaches 2.00 mg/kg. The selenium enrichment of Tan sheep serum, tissues, and organs increased with the increase in dietary yeast selenium addition level (106). Li (107) pointed out that the optimal additional amount of yeast selenium in the diet of Ujumqin sheep is 0.6 mg·kg^−1^·DM^−1^ under natural grazing conditions in the Xilin Gol area; research by Guo and Zhang (108) also confirmed this result.

### Probiotics

The abuse of antibiotics in lamb production is serious. The safe and efficient alternative to antibiotic additives is one of the research hotspots in animal husbandry. Probiotics are safe, efficient, and low-cost and can be used as potential substitutes for antibiotics. *Lactobacillus acidophilus, Streptococcus, Lactobacillus casei*, and *Lactobacillus plantarum* can be colonized in the digestive system of the host, improve the flora structure, inhibit pathogenic microorganisms, and improve the meat production performance of livestock and poultry. Therefore, the addition of probiotics can effectively regulate the gastrointestinal flora of livestock and poultry and has great potential in improving meat quality (109–114). Jia et al. pointed out that *Bacillus licheniformis* and *Saccharomyces cerevisiae* promote growth performance, improve antioxidant capacity and immune function, and are beneficial to fattening lamb rumen fermentation and microbial diversity (115). Bai et al. (116) found that the addition of lactic acid bacteria in the diet can improve the color of the muscle by increasing the proportion of oxidized muscle fibers in Sunite sheep and increase the tenderness of the muscle, thereby improving the quality of the mutton. Du et al. (117) found that the addition of compound probiotics in the diet can improve the structure of Sunite sheep's intestinal flora, metabolites, and blood lipids, thereby improving the quality of mutton. Dou et al.'s (118) research also confirmed that the addition of lactic acid bacteria in the diet can improve the intestinal flora and flavor of Sunite sheep and improve the growth performance, meat quality, and antioxidant capacity.

### Flaxseed

Flaxseed is rich in linseed oil, which is an ideal vegetable oil to increase the PUFA content in mutton. Its LA and ALA content is as high as 70%. Both can reduce blood cholesterol and blood lipids and effectively prevent cardiovascular diseases. Shuang et al. pointed out that feeding flaxseed to lamb can significantly enhance the nutritional properties of n-3 PUFA in body fat, the nutritional value and flavor of tail ester have been improved, and the sautéing processing method has the best effect (119). Zhang et al. pointed out that different forms of linseed oil have no adverse effects on blood lipid metabolism and can increase serum total cholesterol, low-density lipoprotein cholesterol, and high-density lipoprotein cholesterol content, while reducing the concentration of insulin; direct addition of linseed oil will have a certain negative impact on the growth of lamb, which can reduce daily gain and microbial protein concentration; flaxseed sauted grains and linseed oil microcapsule fat powder can improve the production performance of lamb and the function of rumen fermentation, but the effect of microcapsule fat powder is better (120). Liu et al. pointed out that feeding flaxseed increased the abundance of volatile flavor substances in lamb and changed the composition and content of the substances. Flaxseed significantly increases the content of valeraldehyde, trans-2-octenal, and decanal. The results of the electronic nose showed that flaxseed affected the flavor profile of lamb and reduced the odor intensity. The free radical scavenging rate and total antioxidant capacity of lamb in the flaxseed group were significantly higher than those in the control group (121). Wang et al. pointed out that by feeding heated flaxseed, Albas cashmere goat liver DHA content increased significantly, the mRNA expression of ELOVL5 and FADS2 in subcutaneous adipose tissue increased, and the concentration of n-3 PUFA in the rumen also increased (122, 123). Hou et al. (124) pointed out that the addition of linseed in the diet promotes the conversion of glycolytic muscle fibers to oxidized muscle fibers, reduces the diameter and cross-sectional area of various types of muscle fibers, and has a positive effect in improving the color and tenderness of meat.

### Vitamin E

Vitamin E is a fat-soluble vitamin. Because it plays an important role in maintaining the normal reproductive function of animals, it is also called tocopherol. As a natural antioxidant, adding vitamin E to livestock and poultry feed can effectively delay the lipid oxidation of meat products and ensure meat color within a certain period of time. In recent years, researchers have discovered that vitamin E also has a certain regulatory effect on lipid metabolism-related genes (125, 126). Xu et al. pointed out that different doses of vitamin E can significantly increase the mRNA and protein expression of glutathione peroxidase 3 (GSH-Px) and glutathione S-transferase α1 (GSTα1). The increase in the level of antioxidant enzyme gene mRNA and protein, coupled with the increase in antioxidant enzyme activity, is the main reason for the improvement of vitamin E to promote reproductive performance (127). Zhao et al. pointed out that adding 200 IU vitamin E to the diet of each lamb can significantly reduce the content of subcutaneous fat but has no significant effect on the content of intramuscular fat. The composition of fatty acids in meat is one of the main factors affecting the nutrition of meat products. Studies have shown that adding vitamin E to feed increases the content of PUFA. This may be related to the antioxidant properties of vitamin E and its influence on lipid metabolism and metabolism-related genes (128). Research by Liu et al. (129) showed that supplementing 200 IU of vitamin E per day to each Tan sheep can most effectively increase the content of PUFA and CLA while reducing the content of SFA.

### Chinese herbal medicine

Chinese herbal medicine is based on natural animals, plants, and minerals; contains many flavor substances and antibacterial ingredients; and has dual functions of nutrition and medicine (130, 131). It can promote the growth and development of livestock and poultry; improve the quality and flavor of meat; and has no toxic side effects, no residues, and no pollution to the environment, making it an ideal substitute for antibiotics and chemical drugs (132–134). Liu et al. pointed out that the addition of Chinese herbal medicine compound prescription (*Astragalus, Ligusticum wallichii, Atractylodes*, Motherwort, Malt, Hawthorn, *Cinnamon, Magnolia officinalis, Citrus aurantium*, Dandelion) in the diet reduced the crude fat, SFA, and SA content of mutton and increased crude protein, crude ash and dry matter, MSFA, and PUFA, among them, the content of ALA increased by 13.89%, and no heavy metal residues were detected (135, 136). Gao et al. (137) pointed out that the addition of 2% Chinese herbal medicine compound prescription (Licorice, Malt, Fennel, Tangerine peel, White lentils, Cardamom, *Jujube*) in the diet can effectively promote the immunity, growth and development of Bamai sheep, and increase the crude fat content and IMP content in Bamei mutton.

### Lycopene

Lycopene is a carotenoid, mainly found in tomato pulp. This natural plant pigment has strong antioxidant properties, can regulate cell growth and metabolism, and enhances the body's immunity. The polyunsaturated double bond structure of lycopene has the effect of scavenging free radical groups, so that lycopene has strong antioxidant properties. Ma et al. (138) found that the addition of lycopene in the diet significantly reduced the content of inosine, succinic acid, and SA and, at the same time, increased the content of UFA, which can effectively improve the meat quality and flavor of Bamai sheep. Jiang et al. pointed out that the addition of lycopene in the diet can improve the growth of lambs and produce meat with lower fat content and higher PUFA content. At the same time, supplementing with lycopene improved the antioxidant status of lambs and lowered blood lipids. An ideal choice for growing lambs may be 200 mg/kg to prevent environmental stress and maintain normal physiological metabolism (139, 140).

### Other additives

*Alfalfa* and silage are considered important feeds for herbivores, providing abundant feed protein and physically effective neutral detergent fibers. *Alfalfa* saponin is one of the most valuable plant secondary metabolites. Saponins are composed of a fat-soluble core with a steroid or triterpene structure and are amphiphilic. This structure gives saponins membrane-dissolving activity and explains their antibacterial, antitumor, and anti-inflammatory properties in animals. In addition, saponin can act on cholesterol and control lipid metabolism through its ability to bind cholesterol in the intestine and other tissues (110). Liu et al. pointed out that the addition of *Alfalfa* saponins in the diet increased the nutrient digestibility with the increase of *Alfalfa* saponins dosage, especially the average digestibility of dry matter, crude protein, and acid detergent fiber. On average, plasma glucose, TG, and alanine aminotransferase levels decreased with the increase of *Alfalfa* saponin. These results indicate that *Alfalfa* saponins play an important role in increasing nutrient digestibility and plasma metabolite levels (141). Gu et al. found that adding milk substitutes to the diet can significantly increase the daily gain of lambs. Choline supplementation can provide about 60% of the animal's methyl donor requirement. Choline metabolites in the body are highly important for protein, fat, and energy metabolism. When choline is deficient, animals will catalyze methionine to provide methyl groups, leading to potential methionine deficiency. Therefore, the addition of choline in feed may be a potential way to improve animal productivity. Li et al. pointed out that adding 0.25% rumen protective choline (RPC) can promote the growth performance of lamb and improve meat quality. This may be related to the effect on blood lipids and skeletal muscle fatty acid metabolism. However, the beneficial effects of 0.25% RPC supplements need to be verified by more animals. Higher doses, such as 0.75% RPC, are detrimental to live weight gain and ACC expression (90). The types of new feed raw materials and additives are diversified, mostly under the premise of reducing morbidity, ensuring animal health, and saving feed costs, by improving the antioxidant level of mutton sheep, the muscle fibers of mutton can be delayed and thickened, and the odor of mutton can be reduced. However, cost-effective new feed additives are still in the minority. Zhong et al. found that although they have a negative effect on digestion, the addition of green tea polyphenols can improve the growth performance, meat color, tenderness, and shelf life of Ujumqin sheep and reduce the degree of infection of the *Haemonchus contortus* twisted in the intestine (142).

## Conclusion

Inner Mongolia Autonomous Region has a unique geographical advantage in animal husbandry. Since mutton in different regions of Inner Mongolia has its own characteristics, we cannot rationally judge which grassland lamb is better. However, different cooking methods (boiled, roasted, stewed, etc.) put forward different requirements for the tenderness, intermuscular fat content, flavor, and other attributes of lamb. Therefore, the identification of mutton in different regions should be made through two aspects. On the one hand, the local government should regulate the mutton industry chain (including the planning and management of pastures, breeding standards for mutton sheep, slaughtering and processing of mutton sheep, etc.) in accordance with policies as soon as possible; On the other hand, the identification of sheep grazing in different grasslands depends on the whole-process traceability technology and the correlation between different geographical environment characteristics and local meat quality.

Factors such as livestock age, gender, castration, tail docking, feed additives, nutritional level of the diet, grazing and drylot-feeding all affect the quality and flavor of mutton, which are more intuitive than genetic factors. Feed is a key factor in improving the quality and flavor of mutton; the rumen fermentation process and biohydrogenation have a great influence on the composition of intramuscular fatty acids; antioxidant levels of lamb during storage and cooking are also worth investigating. Through the combination of appropriate breeding methods, nutritional control factors and scientific management, the quality and flavor of mutton can be improved, the mutton taste problem of mutton can be fundamentally controlled, and the needs of consumers can be met.

## Author contributions

YL: writing—original draft and writing—review and editing. RL and YY: writing—review and editing. YZ and YH: resources. HW: supervision and funding acquisition. KL: supervision. All authors contributed to the article and approved the submitted version.

## Funding

This research was funded by the Science and technology projects in Inner Mongolia Autonomous Region (No. 2021GG0029) and Central Public-interest Scientific Institution Basal Research Fund (Nos. 1610332022013 and 1610332022009).

## Conflict of interest

The authors declare that the research was conducted in the absence of any commercial or financial relationships that could be construed as a potential conflict of interest.

## Publisher's note

All claims expressed in this article are solely those of the authors and do not necessarily represent those of their affiliated organizations, or those of the publisher, the editors and the reviewers. Any product that may be evaluated in this article, or claim that may be made by its manufacturer, is not guaranteed or endorsed by the publisher.
