# Erratum: Non-genetic factors affecting the meat quality and flavor of Inner Mongolian lambs: a review

**DOI:** 10.3389/fvets.2025.1567490

**Published:** 2025-02-04

**Authors:** Frontiers Production Office

**Affiliations:** Frontiers Media SA, Lausanne, Switzerland

**Keywords:** meat quality, Inner Mongolian, lamb (meat), flavor, non-genetic factors

Following publication, we found a non-genuine email address had been provided for editor Alaa El-Din Bekhit. Following an investigation, which was conducted in accordance with Frontiers policy, we confirmed that the real Alaa El-Din Bekhit was impersonated and did not take any action on this manuscript, and it will therefore be removed from this article. In line with the COPE guidelines on potential peer review manipulation, we conducted a post-publication assessment that concluded that the article meet the standards of publication at Frontiers.

Dr. Matteo Dell'Anno, Review Editor at Frontiers in Veterinary Science, Section Animal Nutrition and Metabolism, conducted a post-publication assessment and concluded that the article is rigorous, scientifically sound, and provides valuable insights to the field.

The publisher apologizes for this mistake.

The original version of this article has been updated.

